# Glycemic Response and Bioactive Properties of Gluten-Free Bread with Yoghurt or Curd-Cheese Addition

**DOI:** 10.3390/foods9101410

**Published:** 2020-10-04

**Authors:** Carla Graça, Joana Mota, Ana Lima, Ricardo Boavida Ferreira, Anabela Raymundo, Isabel Sousa

**Affiliations:** LEAF—Linking Landscape, Environment, Agriculture and Food, Research Center of Instituto Superior de Agronomia, Universidade de Lisboa, Tapada da Ajuda, 1349-017 Lisboa, Portugal; carlalopesgraca@isa.ulisboa.pt (C.G.); joana.mota.p@gmail.com (J.M.); agusmaolima@gmail.com (A.L.); rbferreira@isa.ulisboa.pt (R.B.F.); anabraymundo@isa.ulisboa.pt (A.R.)

**Keywords:** gluten-free bread, dairy products, starch digestibility, bioactivity, celiac disease, irritable bowel disease, preventive diets

## Abstract

The influence of flour replacement by yogurt or curd-cheese additions (from 10% to 20%, *w/w*) on the glycemic response and bioactivity improvements of gluten-free bread was evaluated. Starch digestibility, measured by an in vitro digestion model, was applied to determine the effect on starch fractions. The bread glycemic index was calculated. Bread antioxidant capacity (2,2-diphenyl-1-picryl-hydrazyl-hydrate (DPPH) and ferric-ion-reducing antioxidant power (FRAP) methods) and total phenolic compounds were assessed. Anti-inflammatory properties according to enzymatic matrix metalloproteinase (MMP)-9 inhibitory activity were also studied. Considering the higher level of both dairy products tested (20%, *w/w*) and comparing with control bread results, a reduction of around 35% in the glycemic response of curd cheese bread was achieved, resulting in intermediate index level (glycemic index (GI) 55–69), with yogurt bread still showing a high glycemic index (GI > 70). In terms of bread bioactivity, curd cheese bread expressed better reducing power effects, whereas yogurt bread showed more effective radical-scavenging capacity. An increase in bread phenolic compounds by yogurt (55.3%) and curd cheese (73.0%) additions (at 20%) were also registered. MMP-9 inhibition activity was higher in the dairy bread than in control bread, suggesting an improvement in terms of anti-inflammatory properties. The supplementation of the gluten-free bread by yogurt or curd cheese was shown to be a promising strategy to reduce the glycemic response and to improve the bioactive properties of the bread, that which can contribute to preventive diets of celiac patients and irritable bowel syndrome individuals.

## 1. Introduction

Wheat and gluten-containing products have been associated with a wide range of gastrointestinal disorders. Celiac disease (CD) is the most studied form of gluten intolerance, characterized by a small gut inflammation via an immune response to specific peptides of gliadin, one of the gluten proteins [1]. This inflammation–immune process results in several health problems such as intestinal mucosal damage and villous atrophy [2], leading to malabsorption of macro- and micronutrients [3], which can also lead to small bowel cancer and other associated autoimmune diseases (e.g., diabetes, osteoporosis, and skin disorders) [4].

Furthermore, gluten is not the only triggering component in gastrointestinal disorders. Many other components coexist with gluten in wheat and gluten-related food, such as members of the short-chain carbohydrate group (e.g., fructans), collectively termed FODMAPs (fermentable oligo-, di-, monosaccharides, and polyols), which are associated with several gastrointestinal symptoms in non-celiac gluten sensitivity (NCGS). Irritable bowel syndrome is the common gastrointestinal inflammatory condition associated with non-celiac gluten sensitivity symptoms, characterized by abdominal pain, bloating symptoms, diarrhea, and irregular bowel microbiota [1], strongly impacting the quality of life of these patients.

Accordingly, there has been an increase in demand for gluten-free products, not only due to the greater prevalence of celiac disease but also due to the prevalence of irritable bowel syndrome diseases, since gluten-free grains and derived products tend to be lower in FODPAMs [1].

The recent development of gluten-free foods has focused on research aimed at overcoming the technical challenge of gluten removal from bakery products [5,6], whose nutritional value and health-promotion properties were somehow left behind, being mainly starch-based foods with low protein content, higher in fat levels, with a high glycemic index (GI) [7,8]. 

The glycemic response depends on several intrinsic properties of flour, such as starch grain structure and molecular size, protein and/or lipid content, and the amylose–amylopectin ratio [9]. Previous studies reported that the physical interaction between proteins and starch can decrease the glycemic response by forming a physical barrier that reduces the accessibility of enzymatic attack to starch granules, limiting the degree of starch hydrolysis [8,10], which can be an approach to reduce the glycemic response of gluten-free bread [11].

Additionally, recent works [12,13] showed that some polyphenol compounds (e.g., phenolic compounds, anthocyanins) can reduce digestive enzymes, which can be a strategy to reduce the glycemic index in starchy foods while improving bioactive properties.

In the last decade, the search for anti-inflammatory inhibitors in food has been an important branch of research since it can be a promising strategy for preventive diets.

Matrix metalloproteinases (MMPs) are a zinc-dependent family of endopeptidases, highly involved in the biological human process of connective-tissue remodeling [14]. Specifically, MMP-9 is known to be a key moderator in bowel inflammation processes [15] and carcinogenic processes [16], directly involved in irritable bowel diseases. It is worth noting that chronic inflammatory conditions can degenerate into tumors [17].

Accordingly, the search for new protein-rich sources that contribute to reducing the glycemic index and for bioactive ingredient sources capable of an inhibitory effect against MMP-9 is an important topic of research, since it can contribute to a preventive diet, not only for celiac patients but also for those suffering from irritable bowel disorders. 

Dairy products (DP) are promising protein sources, with recognized nutritional properties and functional benefits. Additionally, they present bioactive compounds associated with human physiological properties, such as antioxidant [18,19], anticarcinogenic, and antimicrobial activities [20].

Furthermore, a few studies showed particular interest in these products, especially those deriving from lactic acid bacteria (LAB) fermentation (e.g., yogurt or cheese), in which secondary metabolites may contribute to an anti-inflammatory activity toward human immune processes, due to the bioactive peptides generated by LAB fermentative activity [21,22,23,24].

Yogurt (Yg) is one of the most nutritious dairy products, widely consumed around the world, due to its functional benefits to the human diet, highlighting it as an alternative ingredient for bakery product supplementation [25,26]. 

Curd cheese (Cc) is a derived dairy product obtained via the manufacturing of soluble whey proteins, characterized by a rich protein source, essential amino acids (e.g., leucine and lysine), and minerals [27], and aromatic amino acids with bioactive properties [28].

This work aimed to study the influence of flour replacement with yogurt or curd-cheese additions (from 10% to 20%, *w/w*) in terms of a reduction in glycemic index and an improvement in the bioactivity of gluten-free bread. The impact on starch performance (pasting properties), determined using heating–cooling microdoughLab assays, was first assessed. To mimic starch digestibility in the human body, an in vitro digestion model was applied. The glycemic index of the gluten-free bread was calculated. The antioxidant capacity of gluten-free bread was evaluated based on scavenging effects (2,2-diphenyl-1-picryl-hydrazyl-hydrate (DPPH)) and ferric-ion-reducing antioxidant power (FRAP). Total phenolic content (TPC) was also determined. The gelatinolytic activity of MMP-9 inhibition was assessed using fluorometric quantification (dye-quenched (DQ) gelatin assay) to assess the anti-inflammatory potential of the gluten-free bread obtained. Linear correlations were tested among pasting properties, starch digestibility, glycemic index, and antioxidant capacity, to acquire additional information about the processes involved.

## 2. Materials and Methods

### 2.1. Raw Materials

Gluten-free bread was prepared according to the bread formulations earlier described [29], using rice flour (Próvida, Pêro Pinheiro, Portugal), buckwheat flour (Próvida, Pêro Pinheiro, Portugal), and potato starch (Colmeia do Minho, Paio Pires, Portugal). 

The fresh plain yogurt (from cow milk) used is a product from LongaVida, Portugal; The yogurt dry extract (11.5%, dry matter) was determined as described earlier [29]. 

The fresh curd cheese (from whey cow milk) used was a commercial product from Lacticínios do Paiva (Lamego, Paiva, Portugal); The dry extract of curd cheese (31.2%, dry matter) was determined as described earlier [29]. 

The dry matter of both dairy products was determined to be considered in the optimization of the gluten-free bread formulations since the replacement was based gluten-free flour basis.

Other ingredients used in bread formulations [29] were commercial saccharose (Sidul, Santa Iria de Azóia, Portugal), salt (Vatel, Alverca, Portugal), dry yeast (Fermipan, Setúbal, Portugal), vegetable fat (Vegê, Sovena Group, Algés, Portugal), and xantham gum (Naturefoods, Lisboa, Portugal) [28,29].

### 2.2. Bread Dough Preparation

Gluten-free bread dough formulations were prepared in according to the procedure earlier described by Graça et al. [29]: the yeast was activated in warm water; dry ingredients were incorporated, well mixed, and kneaded during 10 min; fermentation/leavened (34 °C) of the dough during 20 min at 30 °C was performed, followed by baking at 180 °C during 30 min under convection, according to the breadmaking conditions earlier described [29].

Considering the water coming from each level of the dairy products tested, the water added (WAD) was determined by microdoughLab mixing curves, and the water absorption was calculated according to the procedure earlier described by Graça et al. [29]. Doughs enriched with yogurt or curd cheese were prepared considering incorporations of 20 g and 40 g of each dairy product, which corresponds to 10% to 20%, *w/w*. Replacements were based on gluten-free flour basis, i.e., substituting the dry matter coming from each percentage of yogurt or curd cheese added, on 100 g of flour, as earlier described [26,29]: Ingredients kept constant were salt—1.5 g (0.8%, *w/w*), sugar—2.8 g (1.6%, *w/w*), dry yeast—2.8 g (1.6%, *w/w*), xanthan gum—0.5 g (0.3%, *w/w*), and vegetable oil—5.5 g (3.1%, *w/w*).

The different gluten-free bread formulations tested are presented in Table 1.

The nutritional composition of the different bread formulations, presented in Table 1, was determined in previous work [29], recently published in *Foods*.

### 2.3. Pasting Properties of Gluten-Free Dough

The effect of yogurt and Cc additions (from 10 to 20% *w/w*) on the starch physical behavior of the gluten-free bread dough, in comparison to control dough (CD), was studied by MicrodoughLab measurements (Perten, instruments, Hägersten, Sweden), according to the method earlier described [30], with some modifications. Mixing and heating-cooling curves were applied, according to the following set of conditions: sample homogenization for 30 s, mixing curve at 30 °C for 360 s, heating up from 30 to 95 °C for 390 s, standing at 95 °C for 60 s, cooling down to 50 °C for 390 s, at similar temperature rate (0.17 °C/s). Paddle speed was 63 rpm for running the analysis. MicrodoughLab parameters, that characterize the consistency of the dough during mixing, heating (cooking), and cooling phases were recorded in torque units (mNm) (AACC, 54–60.01): dough development or maximum torque (C1) was reached during mixing at 30 °C, the minimum torque of dough when subjected to mechanical and thermal conditions by heat denaturation of proteins (C2), peak torque of starch gelatinization (C3), cooking stability or minimum torque during the heating period (C4), and final consistency peak torque produced after cooling stage at 50 °C (C5). Tests were performed in triplicates.

### 2.4. In Vitro Starch Hydrolysis

#### 2.4.1. Digestible Starch Fraction

The effect of both dairy products on digestible and resistant starch of the gluten-free bread obtained, in comparison to gluten-free control bread, was evaluated by in vitro starch digestion according to the procedure earlier described [31] and subsequently applied by other researchers [30,32]. Briefly, ground bread crumb samples (100 mg) were dispersed in HCL-KCL buffer (0.1 M; pH 1.5) and incubated (37 °C for 1 h) with pepsin (1 g/10 mL HCL-KCL, 220 U/mL) to prevent protein interactions with starch, simulating the gastric phase. Then, 25 mL of tris-maleate buffer solution (0.1 M, pH 6.9) was added to this mixture to stop the enzyme reaction and to create ideal conditions to initiate the small intestine digestion or pancreatic phase, simulated by adding 5 mL of α-amylase solution (3 U/mL), followed by incubation at 37 °C. Aliquots were collected every 30 min (1 mL, 0–180 min), and the enzymatic reaction was stopped immediately by water-bath boiling (5 min), and kept under cold conditions (until the 180 min of incubation).

Aliquots were treated with 3 mL of sodium acetate buffer (0.4 M; pH 4.75), and 60 μL of amyloglucosidase (3300 U/mL) were added, followed by incubation for 45 min at 60 °C, under constant stirring; volumes were adjusted to 10 mL with distilled water and centrifuged (3.000 *g*/10 min at room temperature, 21 °C ± 2 °C). The supernatant (digestible starch fraction) was used for glucose determination. 

#### 2.4.2. Resistant Starch

Resistant starch was determined according to the methodology earlier described [31] and subsequently applied by other authors [30,32]: The ground bread crumb sample (100 g) was incubated (60 min at 40 °C) with a pepsin solution from porcine gastric mucosa (40,000 U/mL; 1 g/10 mL KCL-HCL buffer), to reduce the protein interference. Subsequently, pancreatic α-amylase (40 mg α-amylase: 200 U/mL) was added and incubated during 16 h at 37 °C, for starch hydrolysis. After hydrolysis, the pellet was isolated by centrifugation and further subjected to digestion with 4 M KOH as described by Goni et al. [31]. This solution was incubated for 45 min at 60 °C, in the presence of amyloglucosidase (3300 U/mL) to hydrolyze the remaining resistant starch to glucose. The pH conditions (pH 4.75) were adjusted according to the enzymatic activity requirements of amyloglucosidase.

Hydrolyzed starch was measured as the amount of glucose released, using the Megazyme GODPOD reagent kit, according to described earlier [30]. Starch was calculated as glucose (mg) × 0.9 (the conversion factor). Tests were performed in triplicates and applied in three bread.

#### 2.4.3. In Vitro Starch Digestion and Estimation of Gluten-Free Bread Glycemic Index

The in vitro digestion kinetics was calculated according to the procedure established earlier [31].

A nonlinear model as expressed by Equation (1) was employed to describe the starch hydrolysis kinetics: C is the concentration at t time, C∞ the equilibrium concentration, k the kinetic constant, and t the time:

(1) $$C=C\infty \;\left(1-e^{-\mathrm{kt}}\right)$$

The hydrolysis index (HI) was obtained from Equation (2), dividing the estimated areas under the hydrolysis curve (AUC 0–180 min) obtained for gluten-free bread and reference food (white wheat bread) [30,31,32]:

(2) $$\mathrm{HI}=\frac{\mathrm{AUC}\;\mathrm{of}\;\mathrm{product}}{\;\mathrm{AUC}\;\mathrm{Reference}\;\mathrm{food}}\;100$$

The estimation of glycemic indices (eGI) was calculated according to Equation (3) [31]:

eGI = (0.549 × HI) + 39.71(3)

### 2.5. Antioxidant and Anti-Inflammatory Activities of the Gluten-Free Bread

#### 2.5.1. Antioxidant Activity

The antioxidant activity of the gluten-free bread was evaluated on ground bread crumb samples (2 g) by preparing methanolic extracts (20 mL of methanol), followed by centrifugation (8000 g/4 °C/20 min) and filtration (0.2 μm filter). The extracts were stored at −4 °C.

The scavenging effect of bread extracts was determined using the DPPH (2,2-diphenyl-1-picryl-hydrazyl-hydrate) methodology [33]: Bread extracts or an acid ascorbic solution (100 μL) were added to DPPH solution (1000 μL) in methanol (90 μmol/L), and the mixture was diluted with methanol (1900 μL). The absorbance (515 nm) was measured after 60 min in a dark room. 

Bread extract reducing power was evaluated applying the ferric ion reducing antioxidant power (FRAP) method [34]: The bread extract or acid ascorbic (90 μL) and methanol (2700 μL) were added to FRAP reagent (2700 μL), and the absorbance (at 595 nm) was measured after 30 min in a shaking water-bath (37 °C). 

Results of scavenging activity and reducing power were expressed as mg ascorbic acid equivalents (AAE) per gram of bread extract. 

The total phenolic content (TPC) of bread extracts was assessed following the method earlier described [35]: The bread extract or gallic acid (150 μL) was added to Folin–Ciocalteu reagent (150 μL, at 0.1 mol/L) and, after 10 min, was well mixed with sodium carbonate (300 μL at 7.5% *w/v*, 300 μL), followed by room temperature dark room incubation (2 h). Absorbance (760 nm) was measured, and TPC results were reported in mg of gallic acid equivalents (AGE) per gram of bread extract.

Tests were performed in triplicates for each antioxidant activity assay, in three bread, to ensure reproducibility of the results.

#### 2.5.2. MMP-9 Inhibition Activity

To evaluate the MMP-9 inhibitory activity of different crumb bread, a buffer-solution extraction was performed: Bread samples (5 g) were added to Tris-HCl buffer solution (30 mL at 100 mM, pH 7.5, ratio 1/6 (*w/v*)), and it was kept stirring for 4 h at 4 °C. After centrifugation (12,000 g for 30 min at 4 °C) (Beckman J2-21M/E), the supernatant was collected and stored (−20 °C). The MMP-9 inhibitory capacity of the yogurt and that of the curd cheese were also evaluated, applying the same procedure described above.

MMP-inhibition activity was tested using the dye-quenched (DQ)-gelatin assay as described before [36]. The fluorogenic DQ-gelatin substrate (Invitrogen, CA, USA) was dissolved in Milli-Q water at 1 mg/mL. All solutions and dilutions were prepared in assay-buffer (50 mM Tris-HCl buffer, pH 7.6, 150 mM NaCl, 5 mM CaCl_2_ and 0.01% *v/v* Tween 20). A black micro-assay plate (Grainer bio-one, 96-well) was used and each well was loaded with 0.1 mM (for a final volume of 200 μL) MMP-9 (Reference: M8945, Sigma-Aldrich, Chemical Company, St Louis, MO, USA), and 80 µL of each bread extract were incubated (30 min at 37 °C). The dairy products and bread extract volume was kept constant (80 µL), equivalent to the following protein content (mg/5 g of dairy product or bread): 8.64 ± 0.40 mg to Yg and 83.0 ± 3.15 mg to Cc, 8.6 ± 1.0 mg to CB, 13.0 ± 0.05 mg to yogurt bread (YgB) and 17.0 ± 0.51 mg to curd cheese bread (CcB). 

Subsequently, remaining MMP-9 catalytic activity quantification, the DQ-gelatin (final concentration of 2.5 μg/mL), was added to each plate well followed by incubation, for 1 h. Since the gelatinolytic activity is present, the DQ-gelatin substrate is hydrolyzed and releases fluorescence (fluorescence measurement conditions: excitation 485 nm/emission 530 nm). To correct possible gelatinolytic activity in the bread extracts, positive (without bread extracts) and negative (without enzyme) controls were also included, and all experimental data were corrected by subtraction the respective negative controls. Tests were performed in triplicates and applied in three bread.

### 2.6. Statistical Analysis

Statistical analysis in the experimental data (average values and standard deviation) (RStudio, Version 1.1.423, Northern Ave, Boston, MA, USA) using variance test in one factor (ANOVA), by Tukey test (post hoc comparisons, at 95% of a significant level), were assessed. A Nonlinear Rheology Model, to fit the experimental rheology data, was applied (Carreau model using TA Instruments\TRIOS software, Waters, Lukens Drive, New Castle, PA, USA). 

## 3. Results and Discussion

### 3.1. Pasting Properties

Starch behavior changes, induced by the addition of yogurt or Cc, were assessed by heating-cooling cycles, to simulate the mixing and baking steps of the breadmaking process.

**Figure 1:**
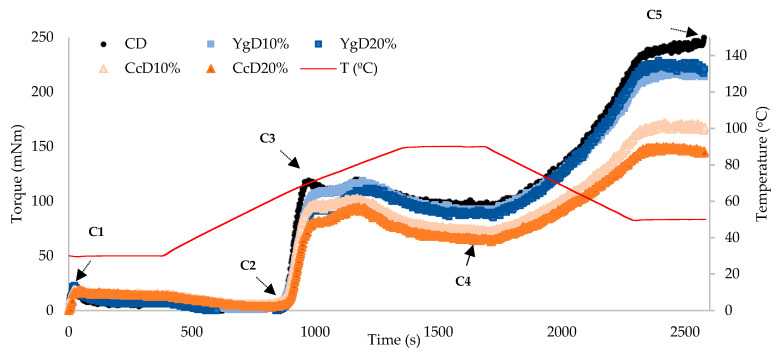
MicrodoughLab heating-cooling curves, expressing the impact of dairy products addition (Yg or Cc) in different amounts (10 and 20%, *w/w*) on starch behavior of gluten-free doughs, compared to control dough: CD—control dough, YgD—yogurt dough, and CcD—curd cheese dough.

Figure 1 clearly shows that the impact of Cc additions on starch performance is much greater than the effect promoted by yogurt incorporations, which can be attributed to the different structure of the proteins involved on the starch-protein matrix, as well as to differences in protein and lipid content [8] between these two dairy products [26]. Protein and lipids content in yogurt and curd cheese bread, at 10 and 20% of addition, varied from 6.9 ± 0.12 g to 8.10 ± 0.20 g and 7.60 ± 0.15 to 9.50 ± 0.10 g for protein content and 5.20 ± 0.84 g to 5.60 ± 0.22 g and 8.90 ± 0.80 g to 10.20 ± 0.30 g for lipids content, in comparison with control bread, 5.34 ± 0.21 g of protein and 4.83 ± 0.29 g of lipids.

During the first seconds of mixing, at constant temperature (30 °C), the torque was very low for all the tested doughs. This result was expected since these doughs comprise mixtures of flours without gluten. Starting the heating stage, at about 60 °C, the systems tended to phase separation, resulting in a further decrease of dough consistency (measured as torque—C2). The values of C2 were too low, namely, for yogurt dough (YgD), expressing a difficulty for the equipment to measure such low torque values, compared to control dough (control dough = 4.3 mNm ± 0.6 mNm). These results suggest that the caseins and exopolysaccharides (EPS) coming from yogurt addition are acting as lubricants with destabilizing effects on the dough matrix [37], reducing considerably the torque under mechanical shear stress. Cc doughs are superimposing the ones from control dough, with mNm values ranging from 6.3 ± 0.6 and 5.7 ± 1.2, showing no significant (*p* > 0.05) differences from control dough.

As the heating phase proceeds (up to 95 °C), starch granules start gelatinizing and take the dominance on torque values, whereas dough consistency increases as a result of starch swelling [38].

Significant impact on starch gelatinization performance was obtained for higher levels of dairy products tested: YgD20%, 101.0 mNm ± 8.5 mNm, and CcD20%, 92.0 mNm ± 1.7 mNm, in which a reduction of around 13% and 21%, respectively, compared to control dough (CD: 116.0 mNm ± 5.0 mNm), was registered. The diminished starch gelatinization observed can probably be attributed to the interaction between starch and protein coming from dairy product additions, reducing the starch availability to swell and break, affecting considerably the dough consistency (in torque values). 

As a consequence of continuous starch granule physical breakdown, due to the mechanical shear stress, further reduction in torque units occurred (C4 value), and at this phase of cooking, the amylase activity takes the dominance, followed by the amylose network stabilization [39]. No significant differences (*p* < 0.05) were obtained on C4 values by yogurt additions, presenting values close to control dough (98.0 mNm ± 2.1 mNm). However, for curd cheese incorporations, significant differences in C4 torque values were noticed, varying from 77.0 mNm ± 3.2 mNm for CcD10% to 65.0 mNm ± 0.6 mNm for Cc20%, compared to control dough, representing a reduction around of 22% and 34%, respectively. 

Subsequently, on cooling (from 95 °C to 50 °C), starch retrogrades and the torque values increase (C5). In terms of final consistency values (C5), slight differences were obtained for yogurt additions, varying from 230 mNm ± 15.1 mNm for Yg10% to 220 mNm ± 16.1 mNm for Yg20%, compared to control dough (250 mNm ± 5.6 mNm). Nevertheless, curd cheese additions promoted a significant impact on C5 values, ranging from 165.3 mNm ± 5.5 mNm for Cc10% to 150.0 mNm ± 1.5 mNm for Cc20%, representing a reduction of around 34% and 40%, compared to control dough values. This considerable reduction in final torque can be attributed to a sort of protective effect of curd cheese proteins on the starch granules hindering gelatinization, reducing the damage of starch granules, and lowering amylose concentrations on continuous phase. Besides, it suggests that the starch granules in the curd cheese dough should be less available to enzymatic hydrolysis than in yogurt doughs. Besides, curd cheese lipids may induce some lubricant effect, further reducing the final consistency.

Linear correlations (*R*^2^ = 0.922) were found between the final consistency and dairy products additions, meaning that the effect on reducing final consistency is proportional to the level of dairy products added (Linear correlations presented in Section 3.5). 

The impact of curd cheese additions on starch behavior was remarkable compared to yogurt incorporations, and it could be attributed not only to the dilution effect on starch but also to the following scenarios:

(i) Insufficient hydration of starch granules resulting from the competition for free water by denatured whey proteins (derived from curd cheese additions [40]);
(ii) Starch-protein interactions, reducing the accessibility of enzymatic attack to starch granules, thus limiting the degree of starch hydrolysis [8];
(iii) Amylose-lipid complexes formed during cooling, lowering the final peak consistency [41].

Results suggested that the different dairy proteins added by yogurt and curd cheese incorporations, promoted different effects on starch performance, mainly on starch gelatinization (C3) and on final dough consistency (C5), being higher for curd cheese incorporations, that probably can have an impact in reducing the glycemic response of bread.

### 3.2. In Vitro Starch Digestion of the Gluten-Free Bread

**Figure 2:**
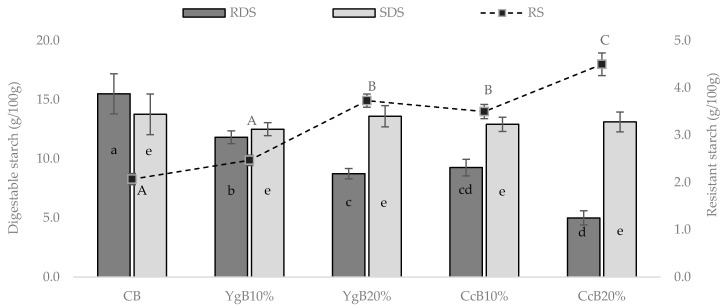
Effect of dairy product additions (10% or 20% *w/w* of yogurt or curd cheese) on starch fractions variation of GFB: rapidly (RDS) and slowly (SDS) digestible starch and resistant starch (RS). Different letters (a–e; A–D) indicate significant statistical differences at *p* < 0.05 (Tukey test).

The influence of yogurt and curd cheese additions to gluten-free bread dough on digestible and resistant starch fractions was evaluated, and the results obtained are presented in Figure 2.

Starch digestibility can be characterized into rapid digestion (RDS)—hydrolyzed within the first 30 min of human digestion; slowly digestion (SDS)—digested within 60–180 min; and resistant starch (RS)—not enzymatically hydrolyzed during human digestion (0–180 min) [42].

From Figure 2, significant (*p* < 0.05) differences on the RDS and RS fractions were achieved, whereas no significant impact was registered in SDS fraction, compared to CB.

Starting from control bread, the dominant starch fraction was RDS followed by SDS and RS. However, upon the addition of yogurt and curd cheese to the dough, this pattern changed, essentially for higher levels (20% *w/w*) tested, where the SDS took the dominance, followed by the RDS and RS. The SDS fraction, being slowly digested, is more desirable than RDS since it promotes a gradual increase in plasma glucose and insulin levels [43].

Figure 2 also shows that a remarkable effect in the RDS reduction was achieved for yogurt and curd cheese additions (10% and 20%, *w/w*), where a significant reduction of about 24% and 44% for yogurt bread and 40% and 67% for curd cheese bread were registered, compared to control bread. Moreover, considerably increases in RS values of around 20% and 80%, and 70% and 110%, respectively, were also obtained.

Yogurt and curd cheese are known to be rich-protein sources (even richer in the latter’s case). Therefore, protein-starch interactions within bread structure, further boosted by the baking process, might affect the digestive enzymes’ accessibility to starch granules, hindering their hydrolytic performance.

These significant decreases of RDS fraction and subsequent increase in RS levels can most probably be linked to the negative impact promoted on starch gelatinization performance (C3). Since the starch was less available to swell and break, probably due to the starch-protein interaction established, the accessibility of enzymatic attack to starch granules was diminished, thus limiting the degree of starch hydrolysis.

Linear correlations were studied between the RDS (*R*^2^ = 0.993) and RS (*R*^2^ = 0.933) results with the protein content of yogurt and curd cheese bread, at 10% and 20% addition (Linear correlations presented in Section 3.5), showing that the variations in both starch fractions are proportional to the increments in protein content by dairy products additions.

Similar results were obtained by other authors [10,11,30], on gluten-free bread enrichment with rich-protein sources.

Additionally, the competition for available water by denatured whey protein should also be invoked to explain these results, lowering the amount of accessible water in the dough system, reducing starch gelatinization due to limited moisture, and impacting negatively on RDS content. 

Therefore, the increase in RS levels can be associated with the diminished catalytic activity of digestive enzymes due to the less accessibility to starch granules, since the starch gelatinization performance was reduced [44].

These results are in line with those obtained in pasting property studies, where a significant effect on starch gelatinization, as well as on final dough consistency values, was obtained, with a major impact by curd cheese addition. Linear correlations (*R*^2^ > 0.900) were found among dairy product additions to the dough, and RDS and RS fractions, as well as the relation between starch fractions and final consistency results (Linear correlations presented in Section 3.5). These findings agree with those recently obtained [30] on the evaluation of the impact of dairy products to improve the glycemic response to wheat bread.

### 3.3. Hydrolysis Kinetics and Estimated Glycemic Index of Gluten-Free Bread

**Figure 3:**
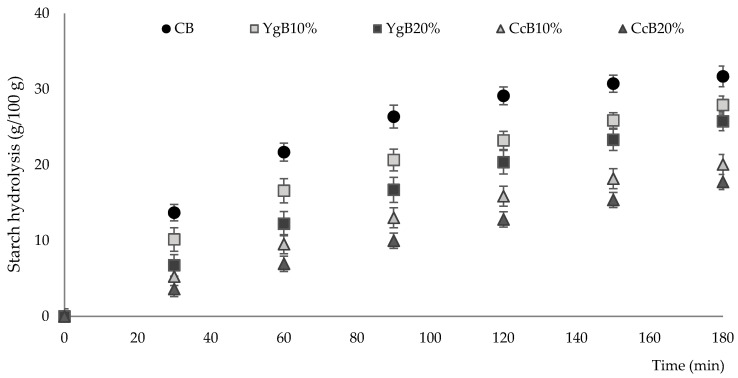
Effect of dairy product additions (10% or 20% *w/w* of yogurt or curd cheese) on starch hydrolysis pattern during in vitro starch digestibility (YgB10% and YgB20%, square symbols) and curd cheese (CcB10% and CcB20%, triangular symbols), compared to control bread (CB, round symbols). Different letters (a–e; A–D) indicate significant statistical differences at *p* < 0.05 (Tukey test).

The gluten-free bread, prepared with the addition of yogurt or curd cheese, and control bread were subjected to in vitro hydrolysis to simulate starch digestibility, and after specific digestion times (30 to 180 min), starch hydrolysis was estimated by the amount of glucose released. Hydrolysis curves and starch hydrolysis vs. time are plotted in Figure 3. 

All digested bread samples exhibited similar behavior, in which the extent of glucose release displayed an almost linear rise between 30 and 60 min of hydrolysis, followed by a gradually diminished as hydrolysis time proceeded (90–180 min).

The influence of yogurt and curd cheese supplementation, in vitro kinetics starch digestion of the bread, was also evaluated, based on primary and secondary parameters derived by fitting the experimental data (*R*^2^ > 0.915) to a nonlinear model (Equation (1)) [31]. These fitted parameters included equilibrium concentration (C∞), kinetics constant (*k*), hydrolysis index (HI), and estimated glycemic index (eGI) and are summarized in Table 2.

Incorporation of yogurt and curd cheese had a significant (*p* < 0.05) influence in C∞ reduction, varying from 28.0 g/100 g to 27.0 g/100 g for yogurt additions, and 20.0 g/100 g to 18.0 g/100 g for curd cheese incorporations, compared to 33.1 g/100 g obtained for CB. Considering the highest levels of yogurt and curd cheese tested (20%, *w/w*), these variations represented a decrease of around 18% and 46% of the equilibrium concentration, respectively, compared to control bread. 

The *k* values that express the enzymatic hydrolysis rate in the early stages decrease substantially by the additions of yogurt or curd cheese: yogurt bread ranging from 0.015 to 0.007 and curd cheese bread varying between 0.007 and 0.003, compared to CB (0.018). Lower values of *k* suggest that the increase of dairy product in the dough is promoting a higher resistance to enzymatic starch hydrolysis, and this may be explained by a starch–protein interactions, limiting starch digestibility.

These results are in line with those obtained on digestible starch fractions, where a significant reduction in RDS and an increase in RS were noticed. Comparable findings were obtained recently by other researchers [45] by “Amala” and plantain enrichment on bread formulations.

Table 2 further shows that the additions of yogurt or curd cheese substantially influenced the starch hydrolysis index (HI), resulting in values considerably lower than control bread. However, a remarkable impact was achieved by curd cheese additions, varying from 53% to 42%, compared to control bread (100%).

These results were reflected in estimated values for the glycemic index, resulting in an intermediate glycemic index for curd cheese bread (GI 55–69), a considerable reduction of about 35% for both dairy products levels tested (10% and 20%, *w/w*). Despite the variations obtained for yogurt levels tested (10% and 20%, *w/w*), the estimated values of the glycemic index are still showing that they maintain a high glycemic response (GI > 70).

### 3.4. Effect of Yoghurt and Curd Cheese Enrichment on Gluten-Free Bread Bioactive Properties

#### 3.4.1. Antioxidant Activity

**Figure 4:**
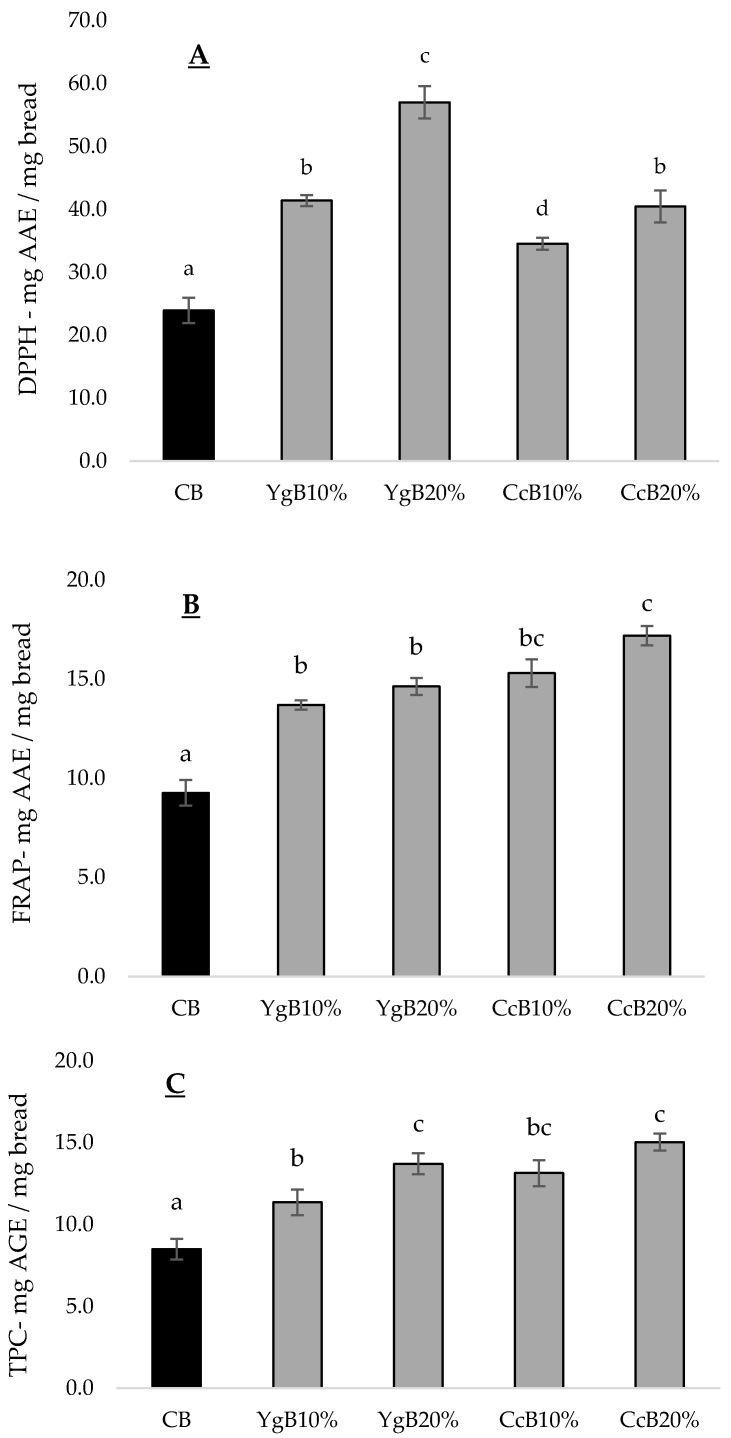
Antioxidant capacity measured with the following methodologies: (**A**)—DPPH, (**B**)—FRAP (both expressed as mg·mg^−1^ ascorbic acid equivalents—AAE) and (**C**)—TPC (total phenolic content, expressed as mg·mg^−1^ gallic acid equivalents—GAE) of fresh breads with 10% (*w/w*) or 20% (*w/w*) yoghurt (YgB10%, YgB20%) or curd cheese (CcB10%, CcB20%), compared to control bread (CB). Different letters indicate significant statistically differences (*p* < 0.05).

The antioxidant capacity (AC) of gluten-free bread enriched with yogurt or curd cheese additions was tested by DPPH and FRAP methods. From Figure 4A,B, it can be observed that both dairy products tested promoted different effects on AC: Yogurt additions showed higher potential to produce bread more effective in radical scavenging capacity (RSC), whereas curd cheese bread expressed better reducing power effects. Compared to CB (DPPH: 24.0 mg·mg^−1^ AAE, FRAP: 10.0 mg·mg^−1^ AAE), the incorporation of the higher level (20%, *w/w*) of yoghurt (DPPH: 57.0 mg·mg^−1^ AAE, FRAP: 15.0 mg·mg^−1^ AAE) and of curd cheese (DPPH: 41.0 mg·mg^−1^ AAE, FRAP: 17.0 mg·mg^−1^ AAE) led to an increase in bread AC, representing an improvement of around 140% and 50% for yoghurt addition and about 71% and 70% for curd cheese incorporation, respectively. The RSC increment on yogurt bread can probably derive from α-tocopherol (vitamin E) since the bioactive properties of this compound contribute to oxidative stability properties [18]. Additionally, it is known that the hydrophobic and aromatic amino acids of the whey protein present bioactive properties [28] that probably can improve the AC of the bread with curd cheese.

Phenolic compounds (PC) are regarded as an important class of secondary metabolites, some of which exhibits health-promoting benefits, including antioxidant activity [19]. The addition of yogurt and curd cheese increased total phenolic compounds (TPC) (Figure 4C): For the highest dairy product level tested (20%, *w/w*) ranged from 13.2 mg·mg^−1^ AGE for YgB, and 15.0 mg·mg^−1^ AGE for CcB, compared to 8.50 mg·mg^−1^ in control bread, representing an increase of 55.3% and 73.0%, respectively. The increase of PC in bread, deriving from dairy products additions, probably contributed to enhancing the antioxidant activity of the bread, expressed as FRAP activity. The linear correlation (*R*^2^ = 0.855) calculated between TPC and FRAP activity supports this relation (Section 3.5, Table 3). Similar results were found by other researchers [10] studying the effect of *Lycium ruthenicum* addition to bread fortification and in vitro digestibility impact.

#### 3.4.2. MMP-9 Inhibition Activity

**Figure 5:**
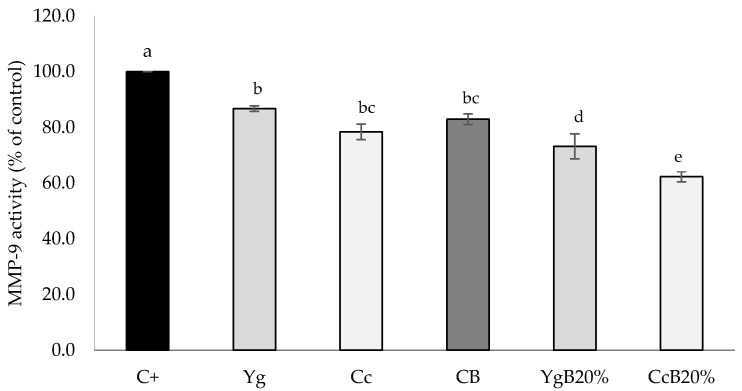
Effect of gluten-free bread extracts on the proteolytic activity of MMP-9 after 60 min of incubation time: Yoghurt (Yg), curd cheese (Cc), control Bread (CB), and bread enriched with 20% (*w/w*) yogurt (YgB20%) or curd cheese (CcB20%). The control (C+) does not inhibit MMP-9. MMP-9 activities were expressed by relative fluorescence as a control percentage (averages of at least. Different letters indicate significant differences (*p* < 0.05), between three replicate experiments (*n* = 3) ± standard deviation of fresh bread.

The impact of yogurt or curd cheese supplementation on gluten-free bread was evaluated by its capacity to inhibit MMP-9 enzyme activity. The DQ-gelatin assay was used to evaluate the anti-inflammatory capacity of bread. The results obtained are illustrated in Figure 5.

The MMP-9 inhibitory capacity of yogurt and curd cheese was assessed individually (Figure 5), in which around 13.3% and 21.6% of inhibition effect were obtained, respectively, compared to control (C+).

Figure 5 also shows that all bread extracts had a significant effect (*p* < 0.05) on MMP-9 activity inhibition, compared to the control (C+). The highest inhibition of MMP-9 activity was obtained in CcB20% (40%), followed by YgB20% (30%), and finally by the control bread (17%). One can suggest that the supplementation of the control bread with each one of the two dairy products tested can be an interesting approach to improve its bioactivity in terms of anti-inflammatory properties, and these properties can probably be further enhanced by the breadmaking fermentation process.

Several studies have been shown the anti-inflammatory properties of dairy products, especially those obtained by fermentation processes, associating them as anti-inflammatory agents on humans’ immune processes [22,24].

These results agree with those obtained in a recent study [46], which showed inhibition of this enzyme by *Limonium tetragonum* extract and are also in line with those reported by other authors [47] based on the inhibition of MMP-9 with phenolic compounds and proteins from cooked soybean.

It can be stated that the MMP-9 inhibition activity exhibited by gluten-free bread with yogurt or curd cheese may constitute an interesting contribution to inflammatory bowel disease preventive diets. Besides, the incorporation of these nutritional dairy products creates an advantage since it can be consumed by the entire population, even by celiac patients, improving their daily diet.

### 3.5. Correlations between Starch Behavior, Glycemic Index, and Bread Bioactivity

According to the results presented along with this research work, a considerable impact on glycemic index reduction by yogurt or curd cheese additions to gluten-free bread was obtained. 

The relationships among pasting properties, starch digestibility, antioxidant capacity, and glycemic index should be considered, to give additional support and consolidate these findings. 

Linear correlations found between pasting properties, starch digestibility, antioxidant capacity, and glycemic index results (*R*^2^ > 0.850) are presented in Table 3.

Strong correlations between the dairy products addition and final consistency (FC) (*R*^2^ = 0.922), RDS content (*R*^2^ = 0.910), RS fraction (*R*^2^ = 0.940), and the glycemic index (*R*^2^ = 0.922) of the breads were obtained. In addition, glycemic index results were also strongly correlated with all these parameters, FC (*R*^2^ = 0.900), RDS (*R*^2^ = 0.940) and RS (*R*^2^ = 0.950). These linear relations reflect a strong correlation between dairy product addition and starch performance changes that, in turn, led to a considerable impact on in vitro starch digestibility and, consequently, on the glycemic index of the gluten-free bread.

According to previous studies [11,12] digestive enzyme inhibition caused by some phenolic compounds was found to be an interesting alternative to maintain a low glycemic index diet, especially for starch-based foods, via inhibition of α-amylase. The possible relationships between the TPC and FRAP activity with glycemic index values were also studied. Linear correlations (Table 3) obtained between glycemic index and TPC (*R*^2^ = 0.900) and glycemic index and FRAP (*R*^2^ = 0.920), suggest that the enrichment in PC and the increase of FRAP activity by the addition of dairy product probably promoted an additional effect on the reduction of the glycemic index of the gluten-free bread. These results agree with those published by other authors [11,12] reinforcing their findings.

## 4. Conclusions

From this study, in which the influence of yogurt or curd cheese supplemented to gluten-free bread was evaluated, to reduce the glycemic response and to improve bioactivity, encouraging results were obtained.

Considering the glycemic index results, the impact of curd cheese addition to dough was greater than those obtained by yogurt additions, significantly reducing the glycemic response to intermediate values of the glycemic index (glycemic index: 55–69).

Improvements in the glycemic response can probably be associated not only to the dilution effect of starch granules and physical interactions between starch and proteins but also to the presence of some bioactive metabolites (e.g., phenolic compounds), derived from the dairy products additions, that we’re able to slow the enzyme’s hydrolysis activity performance while improving the bioactivity of gluten-free bread.

It was possible to improve the bioactivity of bread with both dairy products additions, in terms of antioxidant capacity and phenolic compounds.

The enrichment of gluten-free bread with yogurt or curd cheese resulted in effective inhibition of MMP-9 activity, suggesting that both can be interesting baker’s ingredients to improve the bioactivity of bread in terms of anti-inflammatory properties and possibly anticarcinogenic effects. Nevertheless, further in vitro and in vivo assays to consolidate these anti-inflammatory properties achieved would be tested in future surveys.

The gluten-free bread obtained can give an important nutritional contribution to the celiac and irritable bowel syndrome patients’ daily diet as well as to an inflammation preventive diet strategy.

These findings can be a new window of research to prove the positive impact of dairy product addition to reduce the glycemic index and increase the antioxidant and anti-inflammatory potential of foods.

In summary, the incorporation of yogurt or curd cheese in gluten-free bread showed to be an interesting strategy to improve the glycemic response and anti-oxidative and anti-inflammatory activities, thus contributing to fulfill the needs of the celiac individual’s daily diet.

## Figures and Tables

**Table 1:** Gluten-free bread formulations of control bread (CB), yogurt (YgB), and curd cheese bread (CcB) (the dry extract derived from yogurt (11.5%) and curd cheese (31.2%) additions was considered to replace on flour basis).

* Ingredients (% *w/w*)	CB	YgB_10%_	YgB_20%_	CcB_10%_	CcB_20%_
Buckwheat	16.6	14.0	11.0	12.6	10.0
Rice	25.0	21.0	17.0	20.0	13.0
Potato starch	14.0	11.0	9.0	11.0	8.0
Yogurt/Curd cheese	0.0	10.0	20.0	10.0	20.0
Total water absorption **	37.0	37.5	40.0	39.0	42.0

* Other ingredients kept constant: 7.4%; ** Total water absorption: water added + water originated from Yg or Cc addition, according to the procedure earlier described [29].

**Table 2:** Nonlinear parameters that characterize the in vitro digestibility of gluten-free bread: equilibrium concentration (C∞), kinetic constant (k), hydrolysis index (HI), area under the hydrolysis curve after 180 min (AUC 0–180), and estimated glycemic index (eGI) of control (CB); yogurt (YgB10% and YgB20%) and curd cheese (CcB10% and CcB20%) bread.

Samples	C∞ *	K *	*R* ^2^	AUC 0–180	HI (%)	IG **
CB	33.1 ± 0.9 ^a^	0.0179 ± 0.002 ^a^	0.915	4122.9 ± 61.2 ^a^	100.0 ± 0.0 ^a^	100.0 ± 0.0 ^a^
YgB10%	28.0 ± 0.8 ^b^	0.0153 ± 0.002 ^a^	0.972	3239.6 ± 36.0 ^b^	78.6 ± 1.3 ^b^	82.8 ± 0.7 ^b^
YgB20%	27.0 ± 3.0 ^b^	0.0068 ± 0.001 ^b^	0.984	2766.2 ± 83.0 ^c^	67.1 ± 1.7 ^c^	76.5 ± 0.9 ^c^
CcB10%	20.0 ± 1.2 ^c^	0.0068 ± 0.003 ^b^	0.984	2185.1 ± 28.0 ^d^	53.0 ± 1.0 ^d^	68.0 ± 0.5 ^d^
CcB20%	18.0 ± 0.4 ^c^	0.0029 ± 0.001 ^c^	0.985	1728.3 ± 21.2 ^e^	42.0 ± 0.1 ^e^	62.7 ± 0.1 ^e^

Different superscripts (a, b, c, d, e) indicate significantly statistical differences at *p* < 0.05, (Tukey test), compared to control bread (CB). * C∞ and k were obtained by nonlinear Equation (1): C = C∞ (1 − e^−^^kt^) [31]. ** eGI was estimated by linear Equation (3): eGI = (0.549 × HI) + 39.71 [31].

**Table 3:** Linear correlations were found between pasting properties, starch digestibility, antioxidant capacity, and glycemic index results and dairy product additions (*R*^2^ > 0.850; *p* = 0.05).

Parameters Correlated	Linear Equation	*R* ^2^
FC vs. DP	FC = 218.48 − 3.49 × DP	0.922
RDS vs. DP	RDS = 13.52 − 0.27 × DP	0.910
RDS vs. PBC	RDS = 29.3 − 2.58 × PBC	0.993
RS vs. DP	RS = 2.30 + 0.05 × DP	0.940
RS vs. PBC	RS = - 1.25 + 0.60 × PBC	0.933
RS vs. FC	RS = 7.52 − 0.02 × FC	0.900
GI vs. FC	GI = 23.14 + 0.27 × FC	0.900
GI vs. RDS	GI = 55.32 + 2.68 × RDS	0.944
GI vs. RS	GI = 121.60 − 12.91 × RS	0.952
GI vs. DP	GI = 89.88 − 0.99 × DP	0.922
FRAP vs. TPC	FRAP = 0.60 + 1.26 × TPC	0.850
GI vs. TPC	GI = 134.76 − 5.43 × FRAP	0.900
GI vs. FRAP	GI = 132.80 − 3.97 × FRAP	0.920

**Table legend:** FC—final consistency; RDS—rapidly digestible starch; RS—Resistant starch; DP—Dairy products; PC—protein bread content GI—Glycemic index; FRAP—Ferric ion reducing antioxidant power; TPC—Total phenolic compounds.
